# An Evidence-Based Perspective on “Misconceptions” Regarding Pediatric Auditory Processing Disorder

**DOI:** 10.3389/fneur.2019.00287

**Published:** 2019-03-26

**Authors:** Karin Neijenhuis, Nicole G. Campbell, Martin Cromb, Margreet R. Luinge, David R. Moore, Stuart Rosen, Ellen de Wit

**Affiliations:** ^1^Research Centre Innovations in Care, Rotterdam University of Applied Sciences, Rotterdam, Netherlands; ^2^USAIS, Faculty of Engineering and Physical Sciences, University of Southampton, Southampton, United Kingdom; ^3^Temple Street Children's University Hospital, Dublin, Ireland; ^4^Research Group Healthy Ageing, Allied Health Care and Nursing, Hanze University of Applied Sciences, Groningen, Netherlands; ^5^Department of Otorhinolaryngology, Head & Neck Surgery, University of Groningen, University Medical Center Groningen, Groningen, Netherlands; ^6^Communication Sciences Research Center, Cincinnati Children's Hospital, Cincinnati, OH, United States; ^7^Department of Otolaryngology, University of Cincinnati, Cincinnati, OH, United States; ^8^Manchester Centre for Audiology and Deafness, University of Manchester, Manchester, United Kingdom; ^9^UCL Speech, Hearing & Phonetic Sciences, London, United Kingdom; ^10^Graduate School of Medical Sciences, Research School of Behavioral and Cognitive Neurosciences, University of Groningen, Groningen, Netherlands

**Keywords:** auditory processing disorder, children, diagnosis, consensus, position statement

In the perspective article “Common Misconceptions Regarding Pediatric Auditory Processing Disorder” (1), the authors attempt to rebut five common “misconceptions” of auditory processing disorder (APD), concerned that children with APD may receive inappropriate or limited management. They describe a chasm between increasing research on APD and the scarcity of “*specialized clinics providing diagnosis and management of APD,”* seeing that connection resulting in a failure to translate research into practice. We do not recognize this as a failure. In our opinion, the increasing research interest reflects greater recognition of the importance of evidence-based practice, together with the consolidation of a different perspective—that what is needed is increased collaboration between disciplines, rather than “specialized” audiology APD clinics where diagnosis is based solely on arbitrary audiological test batteries and criteria (2, 3).

There are several definitions of APD. The BSA, 2018 describe APD as being “characterized by poor perception of speech and non-speech sounds. It has its origins in impaired neural function, which may include both the afferent and efferent pathways of the central auditory nervous system (CANS), as well as other neural processing systems that provide “top down” modulation of the CANS. APD impacts on everyday life mainly through a reduced ability to listen, and therefore respond appropriately to speech and other sounds” (3). We share the concerns of Iliadou and Kiese-Himmel [(1); hereafter “the perspective article”] that children with listening difficulties in everyday life deserve proper diagnosis and management in order to prevent or limit their negative impact on academic and social skills and well-being. However, we do not share the perspective that APD has been shown to be a distinct diagnostic entity or that we should focus on traditional auditory testing procedures that lack evidence.

Our purpose here is to argue that the “misconceptions” identified in the perspective article are not misconceptions at all, and arise from the opinions of the authors rather than substantial evidence. We reframe those “misconceptions” in three discussion points: (1) APD as a separate diagnosis, (2) Auditory processing and cognitive skills, and (3) Quality of auditory processing tests.

## APD as a Distinct Diagnosis

According to the perspective article, misconceptions about APD include “APD cannot be diagnosed” and “APD is not a distinct clinical entity.” We share the observation of the perspective article that there is an absence of consensus and a lack of a universal standard. Moreover, there is inconclusive evidence from which it can be concluded that APD can be clearly differentiated from other neurodevelopmental disorders with overlapping symptoms. Most, if not all, cases of childhood APD are characterized by more generally acknowledged learning disabilities (4). Thus, we conclude the opposite: APD cannot be differentially diagnosed at the moment because there are no valid methods to accurately do so.

The perspective article proposes particular criteria to be applied in diagnosing APD, that of “performance at or below 2 SD below the mean in at least 2 validated auditory processing tests that assess different processes in at least one ear, including non-speech sounds.” There are at least 3 problems with these criteria. Firstly, there is no specification of the number of tests that are to be used, and statistically, the more tests performed, the more likely any child is likely to fail two of them. Secondly, there is no specification of the exact tests to be applied, and failure rates are likely to vary greatly from test to test. Finally, although the use of standard deviations to diagnose is a common practice in most clinical fields, the criteria proposed in the perspective article are quite arbitrary. A wide variety of other criteria have been previously proposed, so there is no particular reason to accept these. In fact, Wilson and Arnott (5) showed in a large sample of children that diagnosis rates of APD can range from 7 to 93% depending upon which of previous proposed criteria are applied, even using the same test battery. As a result, they supported “calls to abandon the use of (C)APD as a global label.” One way forward is to use alternative methods to determine cut-off scores, for instance differential or subtractive testing (6) or deriving them individually by sensitivity and specificity estimates, as suggested in the field of language impairment (7).

Along related lines, Vermiglio (8) investigated APD and speech recognition-in-noise disorders in reference to the Sydenham-Guttentag criteria for meeting the definition of a clinical entity; namely that it must have (1) an unambiguous definition, (2) represent a homogenous group with a perceived limitation, and (3) facilitate a diagnosis and intervention. Speech-recognition-in-noise disorders (the most common complaint of individuals presenting for AP assessment) met the criteria but APD did not.

The perspective article also refers to a recently published European consensus paper (9), which in our opinion does not reflect a proper consensus. No methodologically well-designed consensus study has been conducted to support the validity as well as the representativeness of this document. The views summarized therein are essentially those of a position statement first published more than 20 years ago by the American Speech Language and Hearing Association [ASHA; (10, 11)], and subsequently by the American Academy of Audiology [AAA; (12)]. Numerous problems with those position statements have been identified, for example by the British Society of Audiology [BSA; (3, 13)], and by the Dutch Federation of Audiological Centers (14). A rigorous systematic review by Heine and O'Halloran (15) concluded that the APD guidelines of ASHA (11) and AAA (12) should not be recommended because of poor methodological reporting. The effectiveness of the clinical practice guideline of the AAA (12) in supporting assessment and management of APD referrals was recently reviewed by DeBonis (16), using a framework by (17). He concluded that the AAA document (12) does not reflect the current literature, which casts doubt on the relevance of the information provided regarding candidacy, testing, establishing a diagnosis and intervention (16).

## Auditory Processing and Cognitive Skills

The relation between auditory and cognitive skills is discussed under two “misconceptions” (“if APD is a secondary diagnosis, discard it”; “APD reflects cognitive deficits”). As the perspective article indicates, there are many reasons for poor listening. Listening necessarily involves the integration in the brain of bottom-up, auditory “sensory” information with top-down, multimodal “cognitive” information (18). The perspective article seems to claim that it is possible to perform adequate differential diagnosis of APD and thus separate bottom-up from top-down processing skills. This would mean that the population of children with listening difficulties (or “suspected APD”) could be separated into children with “real” APD and children with non-auditory deficits that explain their listening difficulties. We argue that there is insufficient evidence to underpin this claim.

APD is a poorly defined and controversial label that has not become an agreed international standard, despite more than 40 years of attempts. One way forward is to stop considering APD as a “single diagnostic characteristic of the auditory system” as was stated by a leading journal in applied auditory science (Ear and Hearing) (19, 20). Alternatively, the symptoms presented by children with listening difficulties, despite normal audiometry, should be considered in terms of more well-defined, commonly used and almost totally overlapping learning disorders such as developmental language disorder, reading disorders or attention deficit disorder. The crucial point is that, despite the possibility of real auditory problems in these children, current diagnostic and intervention practice for “APD” as advocated by ASHA and AAA is not adequately defined nor based on sufficient evidence to have found general acceptance in the international audiology community.

Another perspective is the ICF Framework (21) that defines a person's health not solely by one's anatomical and physiological features, but also includes personal and environmental factors. In applying this framework to auditory functioning, “APD” could be seen as a disorder at the level of “body functions,” whereas “listening difficulties” can be described as a disability in participation in certain listening contexts [see also (2, 22)]. While the main goal of the framework is to describe human functioning and influencing factors, in this case auditory functioning, the use of diagnostic labels (like “APD”) is not necessary, therefore minimizing controversy. The new BSA Position Statement and Practice Guidance (3) recommended that rather than labeling a person with APD, it is more helpful to describe the listening problems presented, with an emphasis on collaborating with professionals and funders about APD, while also educating them about it. Where a label of APD is necessary to secure support or funding, BSA (3) recommends using only tests that fulfill the criteria of functional specificity, reliability, validity, age-appropriateness and standardization, and providing a clear statement of the diagnostic criteria used.

According to the perspective article, it is a misconception to believe that “the known link between auditory perception and higher cognitive function precludes the validity of APD as a clinical entity.” Tomlin et al. (23) presented an overview of studies investigating the links between cognitive processes and APD and found evidence for a complex interaction between cognitive abilities and auditory processing scores. Two extensive systematic reviews (from 1954 up to May 2015) showed that: (1) The characteristics of children suspected of APD and diagnosed with APD are neither specific nor limited to the auditory modality. Significant differences were found on auditory and visual functioning, cognition, language, reading, physiological measures and brain structure and activity between children referred with listening difficulties and typically developing children (24); (2) Children diagnosed with APD and children diagnosed with developmental language disorder, dyslexia, attention deficit disorder, and learning difficulty shared overlapping characteristics in terms of intelligence, memory, attention and language (4). Based on these results, we argue that there is inconclusive evidence for the existence of a specific auditory deficit in children currently diagnosed with APD.

## Quality of Auditory Processing Tests

For the “misconception” that “We cannot diagnose APD,” the perspective article offered no supportive evidence other than a statement that traditional AP test batteries are appropriate as a gold standard, despite the above evidence and ongoing international debate. This type of reasoning continues in the statement of another “misconception” that “Valid conclusions can be made without testing.”

It seems here the perspective article is suggesting that an APD diagnosis can only be made through the use of the currently available AP tests. However, the question is whether it is even possible to assess AP in a valid way with the currently available tests. As described above, much of the current controversy in the field of developmental APD can be ascribed to arbitrary criteria and test batteries lacking any real scientific underpinnings [see also (5)]. Many commonly used AP tests were developed for adults with identified brain lesions and therefore may not be appropriate for children with developmental APD (3, 25, 26) as many of these tests carry a high language, auditory attention and memory load (3, 25, 26). Inappropriately diagnosing APD in a child with language impairment, for example can delay or hinder access to appropriate language and educational support and undermines the confidence of both referrers and funders.

As we argued above, listening involves the integration of bottom-up and top-down processes. It would be ideal if these processes could be separated using differential diagnostic procedures. However, there is no AP test known with satisfying psychometrics concerning the validity of testing in children that is currently able to differentiate between difficulties in AP and difficulties in language, auditory attention, and/or memory (3, 14). Moreover, one rigorous study that evaluated children on auditory temporal and spectral processing skills found that, when carefully separating sensory and cognitive factors, poor test performance among children was almost exclusively caused by cognitive (attention) factors (18).

In conclusion, we recognize that APD, due to CANS pathology or dysfunction, is a possibility. Also, individuals with hearing loss or identified brain lesions may have additional hearing deficits originating in the central auditory system. We argue however that the APD test battery and criteria proposed in the perspective article are not adequately defined nor based on sufficient evidence for general acceptance by the international audiology community. The suggestion that “specialized” clinics can in isolation diagnose developmental APD using an arbitrary combination of “traditional” APD tests which lack evidence (many of them originally developed for adults with identified brain lesions) is questionable. The focus should not be on the scarcity of “specialized” clinics using these arbitrary tests and criteria but rather on increased collaboration between disciplines involved in diagnostic procedures, given the complexity of the afferent/efferent pathway interaction together with “top down” neural processing systems. It is also time that this comprehensive, multi-/interdisciplinary approach considers the impact of listening difficulties on the child's daily functioning. Developmental APD may contribute to childhood learning difficulties, but its status as a distinct disorder is controversial (3). When dealing with a child with suspected APD it is important, in the first instance, to establish that hearing and middle ear function are within the normal range and likewise that conditions such as Auditory Neuropathy Spectrum Disorder have been ruled out. Other more commonly used and agreed diagnoses of developmental disorders (e.g., developmental language disorder, dyslexia,) should then take diagnostic precedence and importantly any diagnosis of APD should not delay or hinder a child's access to appropriate language, educational, or other support required (3). Where difficulty hearing speech in noise is noted, acoustic modifications to the home or classroom should be considered, together with a trial of remote microphone technology. These should however not be offered as a substitute for language, educational, or other support required. The APD MESHGuide (27), a new evidence-based APD resource commissioned by the British Association of Teachers of the Deaf Foundation, offers a more in-depth discussion of these recommendations. Finally, any analysis of a clinical construct such as APD should be based, wherever possible, on evidence.

## Author Contributions

EdW and ML wrote a first draft. KN completed the first version. NC, MC, DM, and SR contributed by adding and modifying text. We agreed that KN should be first and corresponding author, and that the other authors should be listed in alphabetical order.

### Conflict of Interest Statement

The authors declare that the research was conducted in the absence of any commercial or financial relationships that could be construed as a potential conflict of interest.
